# Electroretinography biomarkers indicate disrupted visual processing in Fragile X syndrome

**DOI:** 10.1186/s11689-026-09684-2

**Published:** 2026-03-27

**Authors:** Qianyi Pu, Naomi Bennett, Tejas C. Sekhar, Mary T. Stanley, Elizabeth Berry-Kravis

**Affiliations:** 1 Department of Pediatrics, Rush University Medical Center, Chicago, IL USA; 2 Department of Neurological Sciences, Rush University Medical Center, Chicago, IL USA; 3 Rush Medical College, 600 S Paulina St Suite 524, Chicago, IL 60612 USA

**Keywords:** Fragile X Syndrome, Visual processing, ERG, Aberrant behavior checklist

## Abstract

**Background:**

Objective physiological biomarkers that index underlying neural circuit dysfunction, such as electroretinography (ERG), are needed in Fragile X syndrome (FXS) research. Fragile X syndrome (FXS) is a neurodevelopmental disorder caused by silencing of the *FMR1* gene and loss of fragile X messenger ribonucleoprotein (FMRP), leading to synaptic dysfunction and prominent sensory processing abnormalities. This study evaluated whether ERG waveform differences are detectable in FXS using a handheld RETeval^®^ protocol while accounting for key technical and physiological determinants of signal variability.

**Methods:**

ERG recordings were obtained during routine clinic visits using the RETeval^®^ system in 24 males with genetically confirmed FXS [aged (mean ± SD) 28 ± 10 years; 19 full mutation, 5 mosaic] and 19 neurotypical male controls [aged (mean ± SD) 26 ± 2 years]. Outcomes included flash and flicker ERG parameters (a- and b-wave amplitudes and time-to-peak; flicker amplitude and time-to-peak). Feasibility was assessed using ERG waveform acquisition and success rates.

**Results:**

Individuals with FXS demonstrated reduced flash b-wave amplitude (β = −6.84 µV; 95% CI [− 12.87 - −0.81]; *p*=.026) and prolonged time-to-peak for flash a-wave (β = 1.79 ms; 95% CI [0.32–3.26]; *p*=.017), flash b-wave (β = 1.10 ms; 95% CI [0.19–2.02]; *p* = .018), and flicker responses (β = 1.68 ms; 95% CI [0.49–2.88]; *p*=.006). Flash a-wave amplitude and flicker amplitude were not significantly different from controls. ERG feasibility was substantially reduced in FXS: participant-level flash acquisition and success were 67% and 46% in FXS versus 100% and 100% in controls, respectively (*p*=.0066 and *p*=.0001). Participant-level flicker acquisition and success were 46% and 38% in FXS versus 95% and 95% in controls (*p*=.0003 and *p*=.0001). No significant laterality effects were observed for waveform parameters or feasibility.

**Conclusions:**

Handheld, light-adapted ERG detected reproducible abnormalities in retinal function in FXS, consistently in reduced flash b-wave amplitude and delayed response timings, supporting altered post-photoreceptor processing as a physiological feature of FXS. Low acquisition and success rates in a routine outpatient clinic workflow indicate feasibility constraints, supporting use of ERG as a context-dependent biomarker for mechanistic studies and interventional trials.

**Supplementary Information:**

The online version contains supplementary material available at 10.1186/s11689-026-09684-2.

## Background

Fragile X syndrome (FXS) is a rare neurodevelopmental disorder caused by an expanded trinucleotide (CGG) repeat in the promoter region of the *Fragile X Messenger Ribonucleoprotein-1 (FMR1)* gene, leading to hypermethylation, transcriptional silencing, and loss or reduction of fragile X messenger ribonucleoprotein (FMRP) expression [1]. FMRP is an RNA-binding protein that regulates dendritic translation, modulates neuronal ion channel function, and supports synaptic plasticity across multiple brain regions [2–10]. Loss of FMRP disrupts these regulatory mechanisms, leading to impaired synaptic development and contributing to the cognitive, behavioral, sensory, and social impairments characteristic of FXS [11, 12].

The clinical phenotype of FXS is characterized by intellectual disability, behavioral challenges such as anxiety and hyperactivity, features overlapping with autism spectrum disorder, and physical features including elongated face and macroorchidism [9, 13, 14]. Among these manifestations, disturbances in sensory processing are particularly prominent, affecting multiple modalities and compounding the cognitive and social difficulties experienced by individuals with FXS [15, 16]. Notably, alterations in visual sensory processing have emerged as a significant contributor to the neurobehavioral phenotype. Impairments in visual processing can lead to delayed sensory-motor development, difficulties with fine motor tasks such as drawing, and deficits in social-emotional recognition [17–19]. Individuals with FXS also show reduced visual attention capacity and diminished sensitivity to contrasts, textures, and motion, consistent with broader sensory integration deficits [16, 19–23].

Visual processing relies on an interconnected network of retinal and cortical pathways. In FXS, abnormalities have been identified not only in the visual cortex and superior colliculus [24–26], but also at the earliest stages of sensory processing within the retina. Retinal neurons express FMRP, and loss of this protein leads to electrophysiological abnormalities detectable by electroretinography (ERG) [27–31]. Perche et al. [32] have demonstrated altered ERG responses in human participants with FXS compared to neurotypical controls, supporting the retina as a biologically plausible site for identifying objective physiological markers of neural dysfunction [32].

Electroretinography is a non-invasive method for assessing retinal function and has been used to identify synaptic transmission abnormalities across a range of neurodevelopmental and psychiatric conditions [33–36]. Recent advances, including the development of handheld devices such as the RETeval^®^ system, have further improved the accessibility of ERG testing. The RETeval^®^ system enables light-adapted ERG recordings without the need for pharmacologic pupil dilation, increasing tolerability for individuals with intellectual disabilities. Its portable, flexible design supports use across diverse clinical settings, with validation established in multiple independent studies [37–39]. Using this device, Perche et al. demonstrated the feasibility of obtaining ERG recordings with FXS under controlled testing conditions [32]. Together, these technological advances suggest that ERG may be used as a practical biomarker for assessing retinal function in populations such as FXS.

Despite these advances, clinical trials in FXS have been hindered by heterogeneous patient presentations and a lack of objective biological endpoints capable of indexing underlying neurophysiological dysfunction or monitoring treatment response [40–43]. In addition, ERG acquisition is sensitive to technical factors such as electrode positioning and to participant tolerance and cooperation, which may be particularly variable in routine outpatient clinic settings. Behavioral features common in FXS may further influence testing feasibility, yet their relationship to ERG acquisition success has not been examined.

Building on prior work, the present study aimed to replicate and extend these findings by using handheld light-adapted ERG recordings to evaluate retinal function in individuals with FXS compared to controls. In particular, we sought to confirm whether abnormalities in ERG parameters are detectable using a pragmatic, clinic-based protocol after accounting for known technical and physiological determinants of signal variability. In addition, we explored whether *FMR1* mutation status and behavioral symptoms, suggested by Aberrant Behavior Checklist–Community (ABC-C) scores factored for FXS (ABC_FX_) scores, are associated with ERG wave parameters as well as acquisition and success. Through this approach, we aimed to further characterize ERG as a physiologically meaningful biomarker of sensory dysfunction in FXS and to address practical barriers to its application in real-world clinical settings.

## Methods

### Participants

Individuals with genetically confirmed Fragile X syndrome were consecutively recruited through the Rush University Medical Center (RUMC) Fragile X Clinic. Clinic coordinators referred participants whom they determined were likely to tolerate handheld electroretinography procedures at the end of their routine clinic visits. *FMR1* mutation status was verified by polymerase chain reaction (PCR) and/or Southern blot analysis and classified as full mutation (> 200 CGG repeats) or mosaic, defined as the presence of both full-mutation and premutation-range (55–200 CGG repeats) alleles.

Neurotypical control participants were recruited as a comparison group and self-reported no history of ophthalmic, neurologic, or systemic disease that would preclude participation. Demographic characteristics, including age, sex, race, pupil diameter, and iris color index, were summarized (Table 1).

**Table 1:** Demographic and ocular characteristics of participants

Characteristic	FXS	Control	*p*-value
Age, years (mean ± SD) [range]	28 ± 10 [14–44]	26 ± 2 [23–31]	0.5723
Race			
Caucasian, n (%)	24 (100)	11 (58)	0.0005
Black, n (%)	0 (0)	0 (0)	-
Asian, n (%)	0 (0)	5 (26)	-
Other, n (%)	0 (0)	3 (5)	-
Sex			-
Male, n (%)	24 (100)	19 (100)	-
Female, n (%)	0 (0)	0 (0)	-
*FMR1* Mutation Spectrum			-
Full Mutation, n (%)	19 (79)	-	-
Mosaic, n (%)	5 (21)	-	-
Pupil Diameter, mm (mean ± SD)	2.46 ± 0.84	2.11 ± 0.66	**0.0014**
Iris Index, (mean ± SD)	1.36 ± 0.11	1.28 ± 0.11	**0.0003**
Electrode Distance, (mean ± SD)	5.2 ± 4.5*	2.70 ± 0.81	**0.0004**

Recordings with electrode placement >4 mm below lower eye lid were excluded from analysis

Participants in either group were excluded if they or a first-degree relative had a known inherited ocular disorder, manifest strabismus, neurologic disease, diabetes mellitus, or were taking medications known to affect retinal function. Additional exclusion criteria included a history of epileptic seizure within the preceding 12 months or any history of significant head or brain trauma or pathology.

Written informed consent was obtained from all control participants and from parents or legal guardians of individuals with FXS. The study was approved by the Rush University Medical Center Institutional Review Board (ORA #05040701) and conducted in accordance with the Declaration of Helsinki.

### Behavioral assessment

The ABC-C is a 58-item caregiver-reported instrument that yields five subscale scores— Irritability, Lethargy/Social Withdrawal, Stereotypy, Hyperactivity/Non-compliance, and Inappropriate Speech —providing a quantitative profile of maladaptive behaviors in neurodevelopmental disorders [44]. The ABC-C was subjected to factor analysis for a large population with FXS, and found to factor into six subscales, with regrouping of items between subscales and addition of a Social Avoidance subscale (ABC_FX_) [45]. The ABC_FX_ scoring algorithm was used for the analyses in this study.

Caregivers completed the ABC_FX_ as part of routine clinical care. For each participant, the ABC_FX_ assessment obtained closest in time to a successful ERG recording was used to best reflect behavioral status during the testing session. For participants with multiple ERG attempts across different clinic visits, the ABC_FX_ scores temporally closest to the successful acquisition were selected. Since behavioral features such as irritability, hyperactivity, and social avoidance may influence cooperation, tolerance of testing, and maintenance of optimal electrode positioning, exploratory analyses were pre-specified to examine associations between ABC_FX_ domain scores and ERG feasibility outcomes. These outcomes included electrode placement quality, ERG acquisition, and ERG success rates, as well as differences in behavioral profiles across *FMR1* mutation subtypes. Analyses involving ABC_FX_ scores were considered exploratory and hypothesis-generating.

### ERG acquisition

All electroretinography recordings were obtained at Rush University Medical Center using a handheld RETeval^®^ device (LKC Technologies, Gaithersburg, MD, USA). Participants with Fragile X syndrome were tested in a quiet clinic room familiar to them, accompanied by a parent or guardian and a clinic coordinator who remained present throughout the procedure to minimize anxiety and facilitate cooperation. Neurotypical control participants were examined in the same testing area under similar ambient lighting conditions (approximately 100 cd·m⁻²), with all testing conducted by the same examiner as for FXS participants.

The ERG protocol followed International Society for Clinical Electrophysiology of Vision (ISCEV) photopic standards [43], with minor modifications to improve feasibility in individuals with intellectual disability. Only light-adapted (photopic) testing was performed. Dark-adapted (scotopic) recordings were omitted because prior pilot testing and published work demonstrated that the required dark adaptation period was poorly tolerated by some individuals with FXS [32]. To support fixation and reduce distress, participants with FXS were permitted to view a short video of their choosing on a smartphone held at eye level behind the RETeval^®^ handset during recording.

Under light-adapted conditions, a single disposable RETeval^®^ adhesive skin electrode strip incorporating active, reference, and ground electrodes was placed on the infraorbital skin of the tested eye. Prior to electrode placement, the skin was cleansed with a 70% isopropyl alcohol wipe and dried to ensure stable adhesion and electrode impedance below 5 kΩ. Electrode vertical position relative to the lower eyelid margin was quantified using infrared images captured by the RETeval^®^ device. Distances were measured manually using Fiji ImageJ software (National Institutes of Health, Bethesda, MD, USA), and pixel measurements were converted to millimeters using the device-reported pupil diameter as an internal calibration reference, allowing estimation of absolute electrode height for each recording.

While electrode placement approximately 2 mm below the lower eyelid margin (RETeval™ Device User Manual Rx Only, 2023) was recommended by the manufacturer, electrode placement ranging from 1 to 4 mm below the lower eyelid margin was used in this study to accommodate inter-individual facial anatomy and behavioral tolerance, consistent with prior ERG studies in Fragile X syndrome [32, 46]. Since electrode vertical position is known to influence ERG amplitude, only recordings obtained with electrode placement ≤ 4 mm were included in quantitative waveform analyses; recordings with placement > 4 mm were excluded from amplitude and time-to-peak analyses but retained for feasibility and acquisition assessments.

The RETeval^®^ device continuously tracked pupil diameter using an infrared sensor and automatically adjusted flash strength (cd·s·m⁻²) to maintain constant retinal illuminance (Trolands) across participants. This approach eliminated the need for pharmacologic pupil dilation and reduced variability related to pupil size, while residual effects of iris pigmentation were accounted for by including the device-derived iris color index as a covariate in statistical analyses.

Each eye was tested sequentially (right eye followed by left eye) using a fixed stimulus order. Flash ERG recordings consisted of 30 single white flashes (85 Td·s, 2 Hz) delivered on an 850 Td white background and averaged to generate a flash waveform. This was followed by a 28.3 Hz flicker stimulus (85 Td·s per cycle) presented on an 850 Td white background and recorded until device-defined precision criteria were met (typically 5–15 s). Recording attempts were automatically terminated if pupil tracking was lost, electrode impedance exceeded 5 kΩ, or the device was unable to achieve the target retinal illuminance.

If an initial recording attempt was unsuccessful, a total of two attempts per eye were permitted within the same session. If bilateral testing could not be completed, participants were re-approached for repeat ERG acquisition during subsequent routine clinic visits. Complete bilateral testing, including electrode placement and recording, typically required 10–15 min.

### ERG measures

**Fig. 1:**
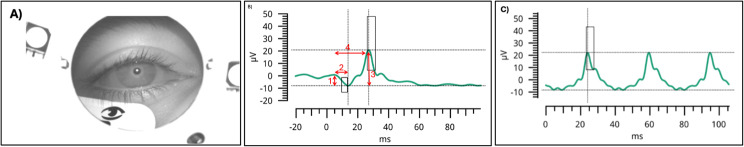
Representative ERG with labeled components, including (**A**) photograph of a participant’s eye illustrating placement of electrode, (**B**) representative flash waveform, (**C**) representative flicker waveform. Labels indicate key waveform parameters: (1) a-wave amplitude, (2) a-wave time-to-peak, (3) b-wave amplitude, (4) b-wave time-to-peak

For flash ERG recordings, the following parameters were extracted from averaged waveforms: (1) a-wave amplitude, defined as the peak-to-trough amplitude from baseline to the a-wave trough; (2) a-wave time-to-peak, defined as the interval from stimulus onset to the a-wave trough; (3) b-wave amplitude, defined as the amplitude from the a-wave trough to the b-wave peak; and (4) b-wave time-to-peak, defined as the interval from stimulus onset to the b-wave peak (Fig. 1). For flicker ERG recordings, the fundamental (first harmonic) amplitude and time-to-peak were extracted from the device-generated waveform.

Recordings lacking clearly identifiable a-wave or b-wave morphology, exhibiting excessive baseline noise or motion artifact, or obtained with electrode placement > 4 mm below the lower eyelid margin were excluded from quantitative waveform analyses and classified as unsuccessful. Only recordings meeting these criteria were included in amplitude and time-to-peak analyses. Representative ERG waveforms and electrode placement images are provided to illustrate recording quality, baseline stability, and typical waveform morphology (Fig. 1).

The RETeval^®^ device recorded pupil diameter and generated an iris color index during each recording. These device-derived measures were retained for use as covariates in statistical analyses.

### Statistical analysis

Statistical analyses were performed using GraphPad Prism (version 10.0; GraphPad Software, San Diego, CA, USA) and Python executed in Google Colab. Demographic and ocular characteristics were summarized as means ± standard deviations for continuous variables and counts with percentages for categorical variables. Group differences in continuous baseline variables (age, pupil diameter, and iris color index) were assessed using Mann–Whitney U tests, and race distributions were compared using χ² tests. Sex was reported descriptively, as all participants were male. *FMR1* mutation subtype (full mutation vs. mosaic) was reported descriptively within the FXS group.

ERG waveform analyses were conducted at the eye level. Left and right eyes were treated as independent observations, as some participants contributed data from only one successfully recorded eye. To account for within-participant correlation arising from bilateral measurements, primary analyses of ERG waveform parameters were performed using linear mixed-effects models with a random intercept for participant. To address potential laterality effects, we compared ERG waveform parameters between left and right eyes within the FXS group (flash a- and b-wave amplitudes and time-to-peak; flicker fundamental amplitude and time-to-peak) using paired Wilcoxon signed-rank tests.

Separate mixed-effects models were fit for each ERG outcome, with fixed effects for group (FXS vs. control), age, pupil diameter, iris color index, and electrode placement. Race was not included as a covariate, as ocular pigmentation reflected by iris index is the physiologically relevant determinant of ERG signal amplitude, and prior work has demonstrated minimal independent effects of race on ERG measures after accounting for pigmentation [47]. Model results are reported as β coefficients, 95% confidence intervals, and two-tailed p-values. Statistical significance was defined as *p* < .05.

ERG feasibility was evaluated using acquisition and success rates at both the participant and eye levels. Acquisition was defined as completion of ERG recording for either the flash or flicker paradigm, regardless of electrode position. Success was defined as obtaining a usable waveform from recordings with acceptable electrode placement (≤ 4 mm below the lower eyelid margin). Inferential comparisons of acquisition and success rates between FXS participants and controls were performed at the participant level using Fisher’s exact test. Left- and right-eye acquisition and success rates were additionally reported and compared using Fisher’s exact test to assess laterality effects. Comparisons involving *FMR1* mutation subtypes were reported descriptively due to the limited sample size of the mosaic subgroup. Eye-level acquisition and success rates were summarized descriptively without inferential testing because of within-participant dependence.

Exploratory behavioral analyses examined ABC_FX_ domain scores across *FMR1* mutation subtypes and between groups defined by ERG electrode placement quality. ABC_FX_ domain scores were summarized using medians with interquartile ranges and compared using Mann–Whitney U tests across mutation subgroups and electrode placement quality groups. Exploratory associations between ABC_FX_ domain scores and ERG parameters were evaluated within the FXS group using participant-level Spearman rank correlations, focusing on flash b-wave amplitude, flash time-to-peak, and flicker time-to-peak. ERG waveform parameters were averaged across left and right eyes when bilateral data were available; otherwise, the single available eye value was used. Given limited sample size and missing data, analyses within *FMR1* mutation subtype groups were considered hypothesis-generating and are presented descriptively.

As multiple ERG outcomes and behavioral domains were examined, no formal adjustment for multiple comparisons was applied. Analyses were pre-specified and exploratory in nature, and effect sizes and consistency across outcomes were emphasized over isolated *p*-values.

## Results

### Participant characteristics

Twenty-four individuals with Fragile X syndrome [aged (mean ± SD) 28 ± 10 years; all male; all Caucasian] and nineteen neurotypical control participants [aged (mean ± SD) 26 ± 2 years; all male; 58% Caucasian, 26% Asian, and 5% Other] completed at least one ERG attempt. Within the FXS cohort, 19 participants were classified as full mutation and 5 as mosaic carriers.

Mean age did not differ significantly between groups (*p* = .57), however FXS participant age ranges were between 14 and 44, while neurotypical participant ages ranged from 23 to 33. Mean pupil diameter during testing was larger in FXS participants compared with controls [(mean ± SD) 2.46 ± 0.84 mm vs. 2.11 ± 0.66 mm; *p* = .0014]. The RETeval^®^-derived iris color index also differed between groups, with higher values observed in FXS participants [(mean ± SD) 1.36 ± 0.11] relative to controls [(mean ± SD) 1.28 ± 0.11; *p* = .0003]. Mean electrode distance was significantly greater in FXS participants than in controls [(mean ± SD) 5.2 ± 4.5 mm vs. 2.70 ± 0.81 mm; *p* = .0004]; recordings with electrode placement > 4 mm below the lower eyelid margin were excluded from waveform analyses. The RETeval^®^ device adjusted flash strength in real time to maintain constant retinal illuminance across variations in pupil size. Demographic and ocular characteristics are summarized in Table 1.

### Electrode placement distribution

Electrode vertical position relative to the lower eyelid margin is summarized in Table 2. Eye-level data are presented descriptively, and no inferential statistical testing was performed due to within-participant dependence.

**Table 2:** Electrode Position Distribution

Group	1 mm, *n* (%)	2 mm, *n* (%)	3 mm, *n* (%)	4 mm, *n* (%)	> 4 mm, *n* (%)	Included in Analysis (≤ 4 mm), *n* (%)	Total # Aquisitions (*N* by eye)	Total # Attempts (by eye)
Control	3 (8)	10 (27)	19 (51)	5 (14)	0 (0)	37 (100)	37 (97)	38
FXS	1 (3)	5 (16)	9 (28)	7 (22)	10 (31)	22 (69)	32(67)	48
Full Mutation	1 (5)	4 (18)	4 (18)	5 (23)	8 (36)	14 (64)	22(58)	38
Mosaic	0 (0)	1 (10)	5 (50)	2 (20)	2 (20)	8 (80)	10(100)	10

Among control participants, electrode placement was consistent. All eye-level acquisitions were obtained with electrode placement ≤ 4 mm below the lower eyelid margin, and 100% of recordings met inclusion criteria for quantitative waveform analysis. The majority of control recordings occurred at 2–3 mm below the lower eyelid margin (78%), reflecting stable electrode positioning during acquisition.

In contrast, electrode placement among FXS participants demonstrated greater variability. Of 48 total eye-level recording attempts in the FXS group, 10 recordings (31%) were obtained with electrode placement > 4 mm and were therefore excluded from quantitative waveform analyses. Overall, 22 FXS eye-level recordings (69%) met the predefined criteria and were included in ERG waveform analyses.

When stratified by *FMR1* mutation subtype, full-mutation participants exhibited a higher proportion of recordings with electrode placement > 4 mm (36%) compared with mosaic participants (20%). Mosaic participants showed a higher proportion of recordings meeting inclusion criteria than full-mutation participants (80% vs. 64%), and all mosaic eye-level acquisitions resulted in successful recordings. Figure 1 depicts a representative successful recording obtained from a participant.

### ERG waveform outcomes

#### Flash ERG

**Fig. 2:**
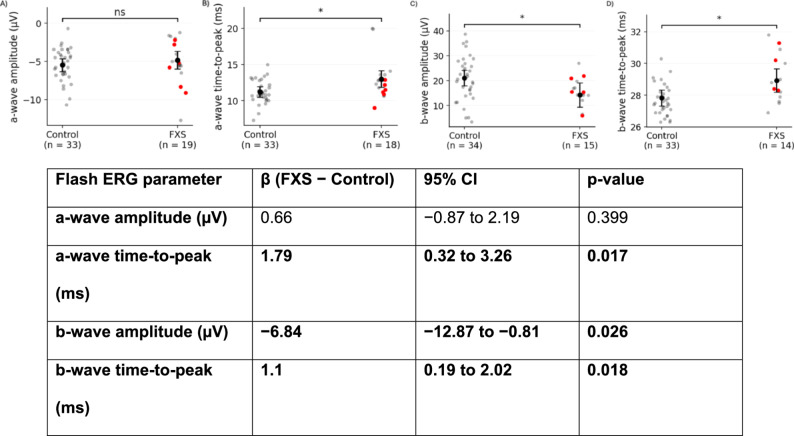
Electroretinography (ERG) results from single-flash stimulation. Comparison of FXS participants and controls for (**A**) flash a-wave amplitude, (**B**) flash a-wave time-to-peak, (**C**) flash b-wave amplitude, and (**D**) flash b-wave time-to-peak

Group differences in flash ERG parameters are summarized in Fig. 2. Outcomes from mosaic individuals in the FXS group was highlighted by red points. In mixed-effects models adjusted for age, pupil diameter, iris color index, and electrode placement, several flash ERG parameters differed between FXS participants and controls.

Flash b-wave amplitude was significantly reduced in FXS participants compared with controls (β = −6.84 µV; 95% CI − 12.87 to − 0.81; *p* = .026), representing the primary electrophysiologic difference observed in flash ERG recordings. Flash a-wave amplitude did not differ significantly between groups (β = 0.66 µV; 95% CI [− 0.87–2.19]; *p* = .399).

Time-to-peak measures demonstrated statistically significant group differences. FXS participants exhibited longer a-wave time-to-peak (β = 1.79 ms; 95% CI 0.32 to 3.26; *p* = .017) and longer b-wave time-to-peak (β = 1.10 ms; 95% CI [0.19–2.02]; *p* = .018) compared with controls.

Visual inspection of individual-level data shows that mosaic participants overlap substantially with full-mutation participants and controls across all flash ERG parameters, without evidence of distinct clustering by mutation subtype. Paired Wilcoxon signed-rank testing comparing left and right eyes within FXS participants revealed no significant laterality effects for flash ERG parameters (a-wave amplitude *p* > .9999; a-wave time-to-peak *p* = .8203; b-wave amplitude *p* = .1094; b-wave time-to-peak *p* = .8438; Supplementary Figure S1).

#### Flicker ERG

**Fig. 3:**
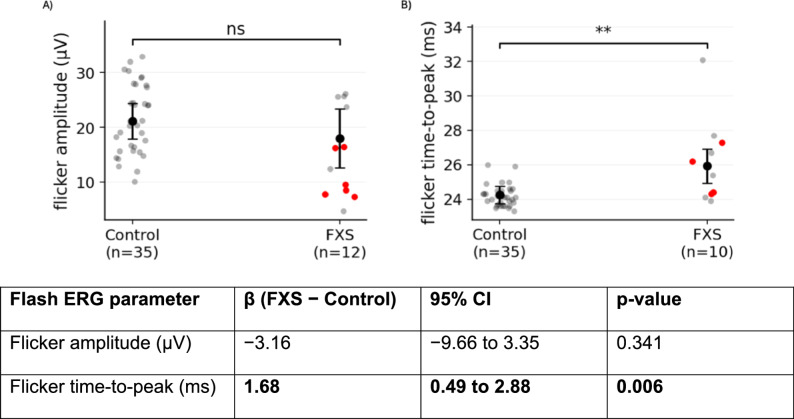
Electroretinography (ERG) results from flicker stimulation. Comparison of FXS participants and controls for (**A**) flicker fundamental (first harmonic) amplitude and (**B**) flicker time-to-peak

Flicker ERG results are summarized in Fig. 3. Flicker amplitude did not differ significantly between FXS participants and controls after covariate adjustment (β = −3.16 µV; 95% CI [− 9.66–3.35]; *p* = .341). In contrast, flicker time-to-peak was significantly prolonged in FXS participants compared with controls (β = 1.68 ms; 95% CI [0.49–2.88]; *p* = .006).

As with flash ERG measures, mosaic participants did not exhibit visually distinct clustering in flicker ERG parameters, and their values overlapped with those of full-mutation participants and controls. No significant left–right differences were observed for flicker amplitude or time-to-peak on paired Wilcoxon signed-rank testing (amplitude *p* = .5469; time-to-peak *p* = .3125; Supplementary Figure S1).

### Aberrant Behavior Checklist-FX (ABC_FX_) scores

ABC_FX_ subscale scores for FXS participants are summarized in Table 3. Scores are reported as medians with interquartile ranges. Median domain scores for the overall FXS cohort were as follows: Irritability 2.5 [0.0–6.0], Hyperactivity 3.5 [2.0–6.0], Lethargy 2.0 [0.0–7.0], Stereotypy 0.5 [0.0–4.0], Inappropriate Speech 3.0 [1.0–5.0], and Social Avoidance 4.0 [0.0–7.0].

**Table 3:** ABC_FX_ subscale scores by *FMR1* mutation subtype

ABC_FX_ Domain (Maximum Score)	FXS Total	Full Mutation	Mosaic	*p*-value
	(*n* = 24)	(*n* = 19)	(*n* = 5)	
Irritability (54)	2.5 [0.0–6.0]	3.0 [0.5–7.5]	0.0 [0.0–1.0]	0.0349
Hyperactivity (27)	3.5 [2.0–6.0]	4.0 [2.0–6.5]	2.0 [1.0–2.5]	0.0836
Lethargy (42)	2.0 [0.0–7.0]	2.0 [0.5–6.0]	1.0 [0.5–4.0]	0.3528
Stereotypy (18)	0.5 [0.0–4.0]	1.0 [0.0–4.5]	1.0 [0.0–3.0]	0.7648
Inappropriate Speech (12)	3.0 [1.0–5.0]	3.0 [1.5–5.0]	4.0 [0.5–5.0]	0.8818
Social Avoidance (12)	4.0 [0.0–7.0]	4.0 [0.0–7.0]	0.0 [0.0–6.0]	0.4032

When stratified by *FMR1* mutation status, full-mutation participants demonstrated higher median irritability scores (3.0 [0.5–7.5]) compared with mosaic participants (0.0 [0.0–1.0]; *p* = .0349), while differences across other ABC_FX_ domains were less pronounced. Given the limited sample size of the mosaic subgroup, mutation subtype analyses were considered exploratory.

### Associations between ERG parameters and ABC_FX_ scores

Associations between ERG parameters and ABC_FX_ domain scores were assessed within the FXS group using participant-level Spearman correlations (Supplementary Table S1). ERG parameters were averaged across left and right eyes when bilateral recordings were available; otherwise, the single available eye value was used, as left–right comparisons showed no significant differences on paired Wilcoxon signed-rank testing (Supplementary Fig. 1). Analyses focused on flash b-wave amplitude, flash b-wave time-to-peak, and flicker time-to-peak. While several moderate-to-strong correlation coefficients were observed, particularly for time-to-peak measures, these associations were inconsistent across domains and did not reach statistical significance.

### ERG feasibility: acquisition and success rates

ERG feasibility was evaluated using acquisition and success rates for both flash b-wave amplitude and flicker amplitude, at both the participant and eye levels (Tables 4 and 5). Acquisition was defined as completion of ERG recording regardless of wave quality or electrode position. Success was defined as obtaining a usable b-wave waveform from recordings with acceptable electrode placement (≤ 4 mm below the lower eyelid margin).

**Table 4:** Acquisition Rate and Success Rate for B-Wave Amplitude at Participant-Level and Eye-Level

Level of Analysis (Total Attempts)	Outcome for B-Wave Amplitude (µV)	Control	FXS Total	Full Mutation	Mosaic‡
Participant-level	Acquisition rate	19 / 19 (100%)	16 / 24 (67%)*	12/19 (63%)	4/5 (80%)
	Success rate	19 / 19 (100%)	11 / 24 (46%)*	8/19 (42%)	3/5 (60%)
Eye-level⁺	Acquisition rate	34 / 38 (89%)	23 / 48 (48%)	16/38 (42%)	7/10 (70%)
	Success rate	34 / 38 (89%)	16 / 48 (33%)	11/38 (32%)	5/10 (50%)

**Table 5:** Acquisition Rate and Success Rate for Flicker Amplitude at Participant-Level and Eye-Level

Level of Analysis (Total Attempts)	Outcome for B-Wave Amplitude (µV)	Control	FXS Total	Full Mutation	Mosaic‡
Participant-level	Acquisition rate	18 / 19 (95%)	11 / 24 (46%)*	7/19 (37%)	4/5 (80%)
	Success rate	18 / 19 (95%)	9 / 24 (38%)*	6/19 (32%)	3/5 (60%)
Eye-level⁺	Acquisition rate	35 / 38 (92%)	19 / 48 (40%)	12/38 (32%)	7/10 (70%)
	Success rate	35 / 38 (92%)	13 / 48 (27%)	7/38 (18%)	6/10 (60%)

### Flash ERG feasibility

At the participant level, acquisition of b-wave amplitude was achieved in all control participants (19/19, 100%) but in only 16 of 24 FXS participants (67%). Similarly, b-wave success was achieved in all control participants (19/19, 100%) compared with 11 of 24 FXS participants (46%). Both acquisition and success rates were significantly lower in the FXS group than in controls (Fisher’s exact test: acquisition *p* = .0066; success *p* = .0001; Table 4).

When stratified by *FMR1* mutation subtype, full-mutation participants demonstrated lower participant-level acquisition (63%) and success (42%) rates compared with mosaic participants (80% and 60%, respectively). These subgroup differences were reported descriptively, as formal statistical comparisons were not performed due to the limited sample size of the mosaic subgroup (*n* = 5).

At the eye level, acquisition and success rates followed a similar pattern, with lower feasibility observed in FXS participants compared with controls. Eye-level acquisition was achieved in 89% of control recordings (34/38) versus 48% of FXS recordings (23/48), and success rates were 89% in controls (34/38) versus 33% in FXS participants (16/48). Eye-level results are presented descriptively and were not subjected to inferential testing because of within-participant correlation.

Within the FXS group, left- and right-eye feasibility was additionally evaluated to assess potential laterality effects (Supplementary Table S2). Acquisition rates were 54% for the right eye (13/24) and 46% for the left eye (11/24), while success rates were identical for both eyes (33%, 8/24). Fisher’s exact testing demonstrated no significant differences between right and left eyes for either acquisition (OR = 1.40, *p* = .7716) or success rates (OR = 1.00, *p* = 1.0000).

### Flicker ERG feasibility

Feasibility for flicker amplitude was lower than for flash ERG, particularly in the FXS group (Table 5). At the participant level, flicker acquisition was achieved in 18 of 19 controls (95%) but in only 11 of 24 FXS participants (46%). Participant-level flicker success was similarly reduced, occurring in 18 of 19 controls (95%) compared with 9 of 24 FXS participants (38%). Both acquisition and success rates were significantly lower in FXS than in controls (two-sided Fisher’s exact test: acquisition *p* = .0003; success *p* = .0001).

When stratified by mutation subtype, full-mutation participants again demonstrated lower flicker acquisition (40%) and success (32%) rates compared with mosaic participants (80% and 60%, respectively). As with flash ERG, subgroup analyses were descriptive due to limited sample size.

At the eye level, flicker acquisition was achieved in 92% of control recordings (35/38) versus only 40% of FXS recordings (19/48), and success rates were 92% in controls compared with 27% in FXS participants (13/48). These eye-level results were summarized descriptively without inferential testing because of within-participant dependence.

Within the FXS group, left- and right-eye feasibility for flicker amplitude was additionally examined to assess potential laterality effects (Supplementary Table S3). Acquisition rates were comparable between right eyes (42%, 10/24) and left eyes (38%, 9/24), with similarly low success rates (29% vs. 25%, respectively). Fisher’s exact testing demonstrated no significant differences between right and left eyes for either acquisition (OR = 1.19, *p* = 1.000) or success rates (OR = 1.24, *p* = 1.0000).

### Behavioral correlates of electrode placement quality

**Fig. 4:**
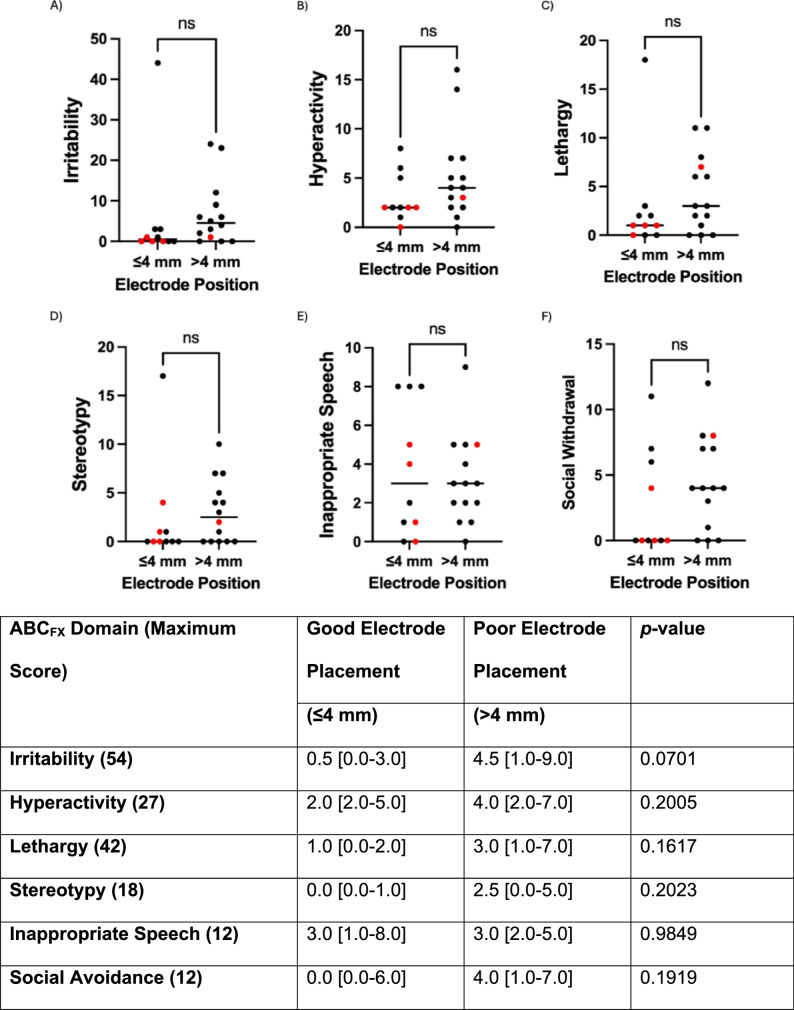
Electrode placement and ABCFX domains, including irritability (**A**), hyperactivity (**B**), lethargy (**C**), stereotypy (**D**), inappropriate speech (**E**), social withdrawal (**F**)

Since acceptable electrode placement is a prerequisite for successful ERG waveform acquisition, we next examined whether behavioral characteristics were associated with electrode placement quality. ABC_FX_ domain scores were compared between participants with good electrode placement (≤ 4 mm) and those with poor electrode placement (> 4 mm). Participants with good electrode placement tended to demonstrate lower behavioral symptom burden across multiple domains. Irritability scores were lower in the good-placement group compared with the poor-placement group (0.5 [0.0–3.0] vs. 4.5 [1.0–9.0]), approaching statistical significance (*p* = .0701). Other domains, including Hyperactivity, Lethargy, Stereotypy, and Social Avoidance, were numerically lower with good placement but did not reach statistical significance (all *p* > .16). Inappropriate Speech scores were similar between groups (*p* = .9849) (Fig. 4).

## Discussion

In this study, we evaluated the feasibility and electrophysiologic characteristics of handheld, light-adapted electroretinography in individuals with Fragile X syndrome using a pragmatic, clinic-based protocol. After explicitly accounting for technical and physiologic factors known to influence ERG recordings—including electrode vertical position, age, pupil diameter, and iris color index—we observed reproducible differences in select ERG parameters between individuals with FXS and neurotypical controls. Specifically, flash b-wave amplitude was reduced in FXS, and both flash and flicker responses demonstrated significant prolongation of time-to-peak. Concurrently, feasibility analyses revealed substantially lower acquisition and success rates in FXS compared with controls, with behavioral tolerance and electrode representing critical practical barriers to data yield in routine clinical settings.

### Comparison with prior ERG studies in FXS

These findings both replicate and extend prior work examining electroretinographic abnormalities in Fragile X syndrome. Perche et al. [32] reported significantly reduced light-adapted flash b-wave amplitude and flicker amplitude, along with prolonged flicker time-to-peak, in males with FXS using the same handheld RETeval^®^ device in a highly controlled, home-based testing environment. Consistent with that study, we observed a significant reduction in flash b-wave amplitude in FXS participants, supporting the reproducibility of inner retinal response magnitude abnormalities across independent cohorts.

In contrast to Perche et al., flicker amplitude differences did not reach statistical significance in our adjusted models, whereas flicker time-to-peak remained significantly prolonged in FXS. Several methodological differences likely contribute to this divergence. Perche et al. did not explicitly adjust ERG analyses for iris pigmentation, pupil diameter, electrode vertical position, or within-participant dependence, whereas these factors were incorporated as covariates and random intercepts in our mixed-effects models to account for known sources of signal variability and eye-level correlation [46, 47]. In addition, our recordings were obtained in a routine outpatient clinic setting, rather than a home environment, and were characterized by substantially lower acquisition and success rates in the FXS group. The reduced number of analyzable eye-level recordings may have limited sensitivity for detecting amplitude differences under flicker conditions, while preserving sensitivity to timing abnormalities.

Recently, Hasan et al. (2025) expanded the ERG literature in *FMR1*-related conditions by examining individuals with FXS, premutation carriers, and controls using the RETeval^®^ system [46]. That study reported significantly reduced b-wave amplitude and flicker amplitude, with the greatest reductions observed in FXS males, followed by FXS females, premutation carriers, and controls. In addition, b-wave time-to-peak was prolonged in individuals with FXS, particularly in FXS males, compared with FXS females, premutation carriers, and controls.

Our findings align with this broader pattern, particularly with respect to b-wave amplitude abnormalities and delayed response timing, although Hasan et al. reported more pronounced differentiation across mutation subgroups, but the premutation subgroup studied by Hasan et al. would be very different from the FXS group while mosaics would be expected to be more similar. In our cohort, electrophysiologic waveforms were broadly similar across mutation subtypes; however, formal subgroup comparisons were limited by the small number of mosaic participants. Hasan et al. further emphasized that ERG metrics did not correlate with peripheral molecular or behavioral measures, suggesting that ERG captures a physiological retinal phenotype that may be dissociable from clinical symptom severity.

While our study did not formally examine molecular correlates, we explored behavioral characteristics and *FMR1* mutation subtypes in relation to ERG waveform parameters as well as ERG feasibility. ERG waveform parameters did not show significant correlations with ABC_FX_ domain scores, consistent with observations reported by Hasan et al. In contrast, behavioral factors—particularly irritability—were associated with electrode placement quality and thus ERG acquisition and success.

Taken together with prior work, these findings indicate that b-wave amplitude and b-wave time-to-peak abnormalities are consistently observed across studies of FXS, despite differences in testing environment, analytic adjustment, and feasibility. This convergence supports the interpretation that cone-driven retinal signaling alterations represent a reproducible neurophysiological feature of FXS, while highlighting the importance of behavioral tolerance and electrode stability as key determinants of data yield in clinical settings.

### Interpretation of ERG abnormalities

The flash b-wave, which primarily reflects ON-bipolar cell depolarization with contributions from Müller cells, serves as an index of post-photoreceptor signal amplification following a light stimulus [40, 41, 48]. In this study, the reduction in flash b-wave amplitude observed in individuals with FXS suggests diminished signal gain or reduced synaptic efficiency within inner retinal circuits. In parallel, the prolongation of b-wave time-to-peak indicates delayed signal integration or transmission within these same post-photoreceptor pathways. Considered together, these findings are consistent with altered bipolar cell–mediated processing rather than isolated abnormalities in photoreceptor activation.

At the level of photoreceptors, flash a-wave amplitude was preserved, whereas a-wave time-to-peak was prolonged in individuals with FXS. Because the light-adapted a-wave primarily reflects cone photoreceptor hyperpolarization, this dissociation suggests that the magnitude of cone responses remains largely intact, while the kinetics of phototransduction or early cone signaling may be slowed [42]. Such a pattern is more consistent with subtle delays in response timing or recovery rather than a reduction in photoreceptor signal strength.

Under high-frequency flicker stimulation, ERG responses are dominated by postreceptoral ON- and OFF-pathway activity, with minimal direct contribution from photoreceptors [49]. In this context, flicker amplitude was relatively preserved in adjusted models, whereas flicker time-to-peak was significantly prolonged in FXS. Preservation of flicker amplitude suggests that cone-driven retinal circuits retain the capacity to sustain repetitive responses, while delayed time-to-peak is consistent with slower temporal integration or recovery within postreceptoral pathways, most plausibly at the level of bipolar cell processing.

Mechanistically, these physiological findings align with prior work implicating synaptic inefficiency in FXS. Loss of fragile X messenger ribonucleoprotein is associated with immature dendritic spine morphology and impaired synaptic plasticity, which may reduce synaptic efficiency and slow signal integration across neural circuits, including within the retina [31, 47]. Although these mechanisms cannot be directly inferred from ERG alone, the combined pattern of preserved photoreceptor response magnitude, reduced post-photoreceptor signal gain, and delayed response timing is consistent with altered retinal signal amplification and synaptic processing rather than gross defects in phototransduction.

### Feasibility and technical considerations

Despite demonstrating reproducible electrophysiologic differences between groups, ERG feasibility was substantially reduced in individuals with FXS in this clinic-based study. At the participant level, acquisition and success rates for flash b-wave amplitude were 67% and 46%, respectively, while flicker acquisition and success were further reduced to 46% and 38%. No laterality effects were observed for feasibility. These feasibility rates are notably lower than those reported by Perche et al. [32], in which approximately 75% of individuals with FXS provided usable flash ERG data and 85% completed flicker ERG in at least one eye [32].

Several methodological differences likely account for this discrepancy. Perche et al. conducted testing in a home-based environment. In contrast, the present study was performed at the end of their routine outpatient clinic setting, and participants were referred by clinical coordinators based on their judgement of amenability.

Electrode positioning emerged as an important technical contributor to reduced ERG feasibility in FXS. Compared with controls, individuals with FXS demonstrated substantially greater variability in electrode vertical position, with nearly one-third of FXS eye-level recordings exceeding the predefined placement threshold (> 4 mm below the lower eyelid) and therefore excluded from analysis, whereas no control recordings exceeded this limit (Table 2). This pattern was most pronounced in participants with full mutation FXS and less evident in mosaic participants, paralleling differences observed in overall acquisition and success rates. However, interpretations regarding mutation subtypes are limited by the small size of the mosaic subgroup. Although electrode placement was not formally modeled as a predictor of success, these distributions indicate that maintaining stable electrode positioning represents a key technical challenge in FXS, particularly in clinic-based settings, and likely contributes to reduced data yield alongside behavioral tolerance factors.

### Behavioral factors and ERG feasibility

Behavioral characteristics were recognized in advance by testing personnel as a potential factor influencing electrode placement stability and ERG feasibility and were therefore examined exploratorily within the FXS cohort. Within this group, irritability scores were lower among participants who achieved acceptable electrode placement compared with those whose recordings exceeded the placement threshold, although this association did not reach conventional statistical significance. Other ABC_FX_ domains, including Hyperactivity, Lethargy, Stereotypy, Inappropriate Speech, and Social Withdrawal, were not significantly associated with electrode placement quality.

Consistent with these feasibility-related observations, irritability scores also differed by *FMR1* mutation subtype, with mosaic participants demonstrating significantly lower irritability compared with full-mutation participants. Interpretation of mutation subtype effects is limited by the small size of the mosaic subgroup (*n* = 5); however, this behavioral profile parallels the higher acquisition and success rates observed in mosaic participants. At the same time, the absence of significant correlations between ERG waveform parameters and ABC_FX_ domain scores within the subset of successful recordings suggests that behavioral symptoms may be more informative for feasibility-related outcomes (electrode stability and data yield) than for variation in ERG measures once adequate recordings are obtained.

### Implications

Despite the substantially reduced acquisition and success rates observed in this clinic-based cohort, the present findings support the relevance of flash b-wave measures as a candidate electrophysiologic biomarker in Fragile X syndrome. These results indicate that handheld ERG acquisition using the RETeval^®^ system may not yet be scalable for routine outpatient clinical use in individuals with FXS, given the low success rates observed under awake, clinic-based testing conditions. Across this study and prior independent cohorts, reductions in flash b-wave amplitude and prolongation of b-wave time-to-peak emerge as reproducible features of FXS, indicating altered post-photoreceptor retinal processing.

From a translational perspective, limitations in feasibility and scalability under awake clinic-based conditions do not diminish biomarker validity, as ERG is routinely performed under sedation or general anesthesia when clinically indicated, with well-characterized and interpretable effects on waveform amplitude and timing that can be accounted for analytically [50–52]. Moreover, prior work using the same handheld RETeval^®^ system demonstrated substantially higher acquisition success when testing was conducted in a controlled, home-based setting dedicated to ERG acquisition, underscoring that feasibility is strongly dependent on acquisition context rather than on the device itself [32].

In this context, the identification of flash b-wave abnormalities as a reliable marker of retinal circuit dysfunction remains relevant for both mechanistic investigations and future interventional trials, where ERG may serve as an objective physiological marker of neural circuit function and treatment response rather than as a diagnostic tool.

### Limitations

Several limitations should be considered when interpreting these findings. First, ERG feasibility was reduced in this clinic-based cohort of individuals with FXS, resulting in lower acquisition and success rates compared with prior home-based studies. Reduced data yield, particularly under flicker conditions, limited the number of analyzable recordings and may have reduced sensitivity for detecting amplitude differences in some paradigms.

In addition, baseline differences between FXS and control participants were observed, including differences in age distribution, pupil diameter, iris color index, and electrode placement. These variables were explicitly accounted for in adjusted analyses, and pupil size was continuously tracked and automatically adjusted for by the RETeval^®^ system to maintain constant retinal illuminance.

However, residual technical variability related to movement, blinking, or brief loss of alignment cannot be fully eliminated in awake testing, especially in neurodevelopmental populations. In particular, irritability scores were lower in participants with good electrode placement compared with those exceeding the placement threshold, approaching statistical significance. Although this association did not meet conventional significance thresholds, the consistent directionality suggests that irritability may be an important behavioral factor affecting electrode stability and, in turn, ERG feasibility. Larger studies will be required to determine the independent contribution of behavioral symptoms to electrode placement and data yield using multivariable or longitudinal approaches.

Subgroup analyses by *FMR1* mutation status were limited by the small number of mosaic participants, precluding definitive conclusions regarding electrophysiologic differences across mutation subtypes. Finally, this study was cross-sectional and did not include longitudinal or treatment-related assessments; therefore, the sensitivity of ERG measures to within-subject change over time or therapeutic intervention cannot be directly evaluated. Despite these limitations, the consistency of flash b-wave amplitude and timing abnormalities across independent cohorts supports the biological relevance of these findings and motivates future studies using optimized acquisition conditions, larger samples, and longitudinal designs.

## Conclusions

In summary, this study demonstrates that handheld, light-adapted ERG can detect reproducible abnormalities in retinal function in individuals with Fragile X syndrome within a pragmatic clinical setting, while also delineating the technical and behavioral factors that constrain feasibility. Reduced flash b-wave amplitude and prolonged b-wave time-to-peak emerged as consistent electrophysiologic features of FXS, reinforcing evidence for altered post-photoreceptor retinal processing across independent cohorts. At the same time, the low success rates observed in this clinic-based cohort indicate that the handheld RETeval^®^ system may not yet be scalable for routine outpatient clinical use in individuals with FXS. However, these limitations in data yield does not diminish the translational relevance of ERG. Together, these findings highlight the importance of optimizing acquisition conditions and accounting for behavioral tolerance and electrode stability when deploying ERG in neurodevelopmental populations. Importantly, the reproducibility and physiological specificity of flash b-wave abnormalities support their potential utility as objective biomarkers for monitoring neural circuit function and treatment response in future interventional studies, rather than as diagnostic tools.

## Supplementary Information

Supplementary Material 1.

## Data Availability

The data supporting the main findings of this study are available within the manuscript. Additional data generated during the study that are not included in the manuscript are available from the corresponding author on reasonable request.

## Abbreviations

FXS: Fragile X Syndrome
FMRP: Fragile X messenger ribonucleoprotein
FMR1: Fragile X Messenger Ribonucleoprotein-1
ERG: Electroretinography
ABC-C: Aberrant Behavior Checklist-Community
ABC_FX_: The ABC-C with modifications for FXS participants (regrouping of items between subscales and addition of a Social Avoidance subscale)
RUMC: Rush University Medical Center
PCR: Polymerase chain reaction
ISCEV: International Society for Clinical Electrophysiology of Vision
