# Supplementary material for: Electroretinography biomarkers indicate disrupted visual processing in Fragile X syndrome
Source: J Neurodev Disord. 2026 Mar 27;18:27. doi: 10.1186/s11689-026-09684-2 (PMC13188770; doi:10.1186/s11689-026-09684-2)
Supplement: Supplementary file 1 — Supplementary Material 1. [file 11689_2026_9684_MOESM1_ESM.pptx]

## Slide 1
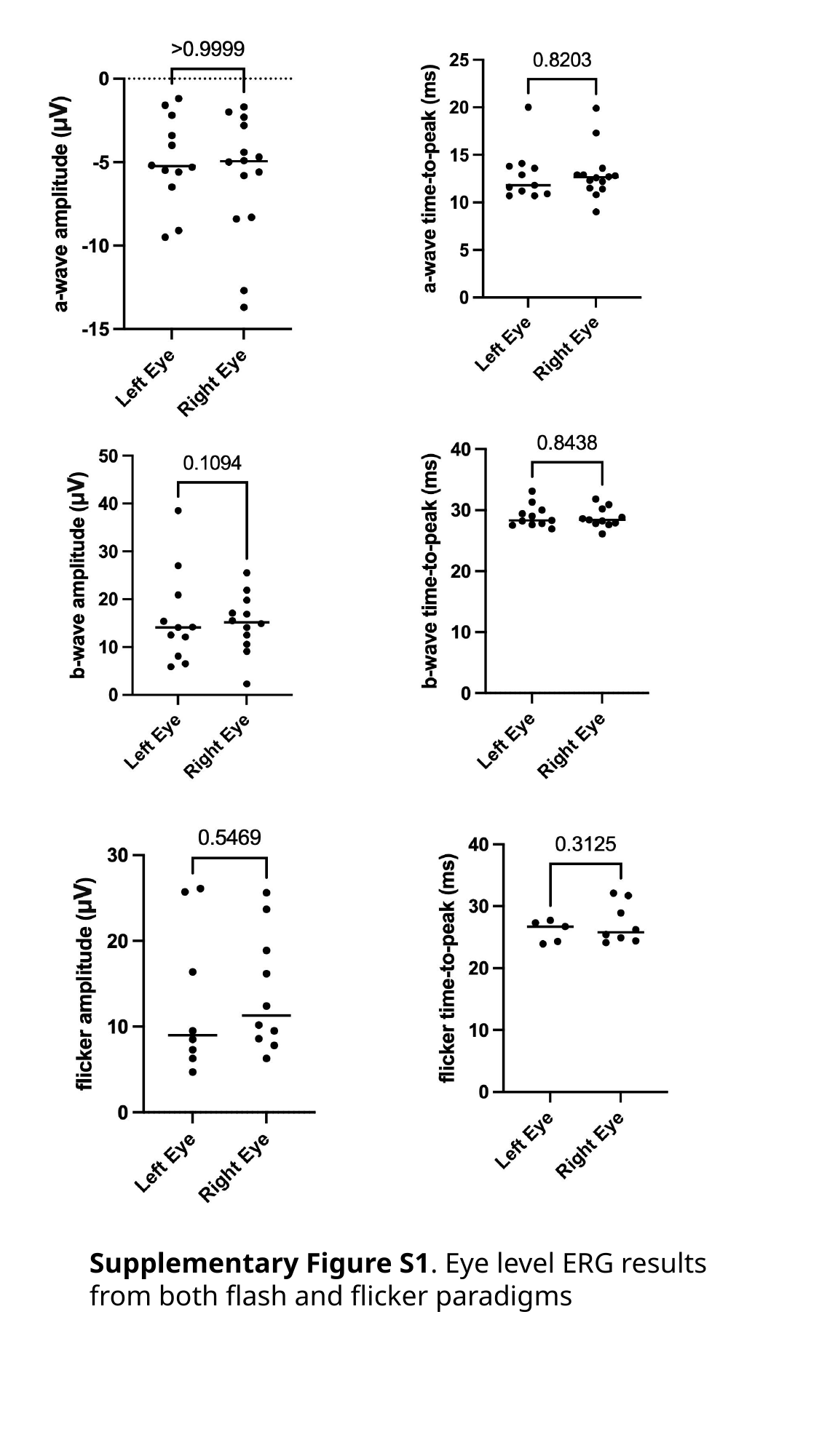

Supplementary Figure S1. Eye level ERG results from both flash and flicker paradigms

## Slide 2
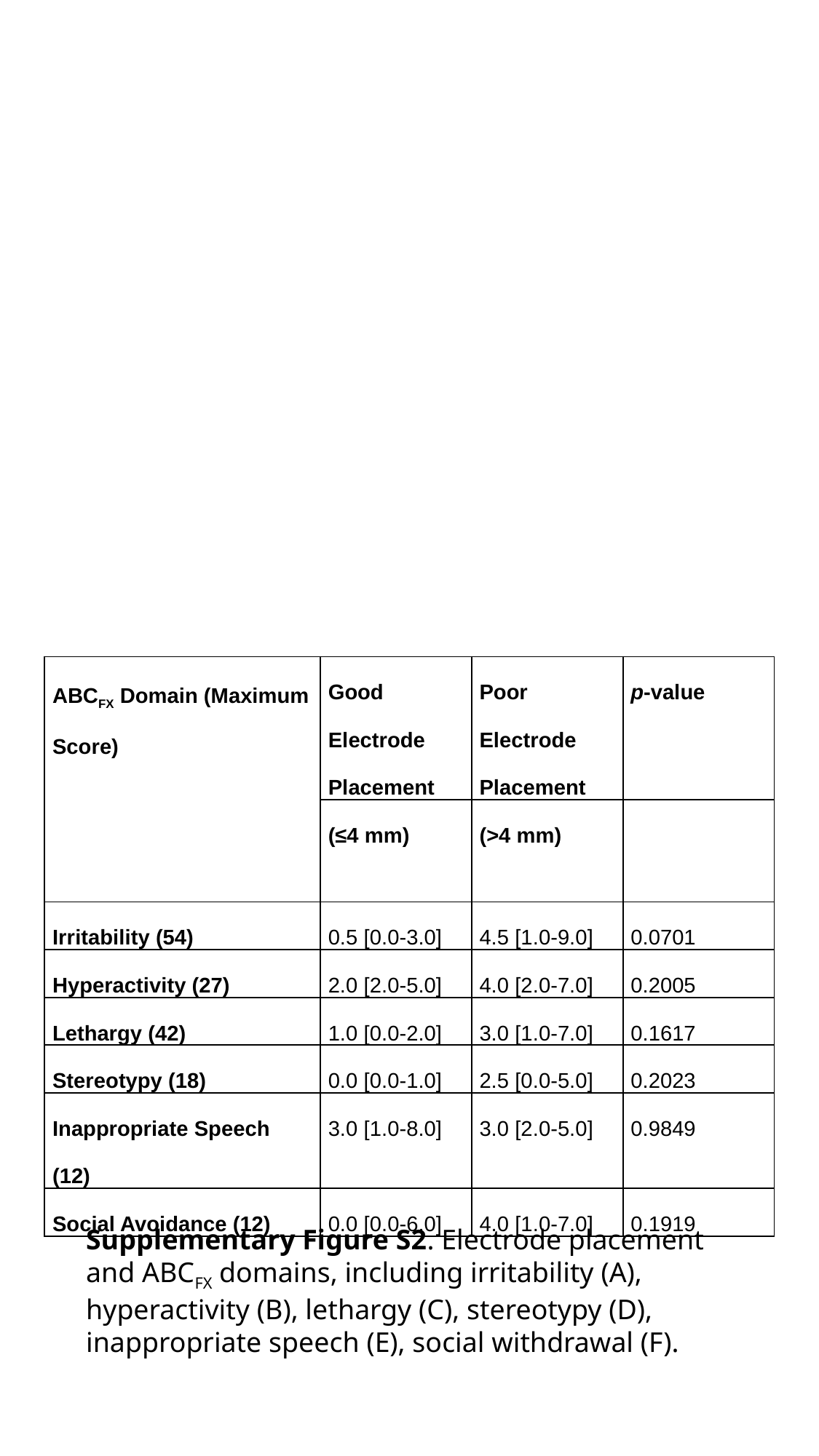

| ABCFX Domain (Maximum Score) | Good Electrode Placement | Poor Electrode Placement | p-value |
| --- | --- | --- | --- |
| | (≤4 mm) | (>4 mm) | |
| Irritability (54) | 0.5 [0.0-3.0] | 4.5 [1.0-9.0] | 0.0701 |
| Hyperactivity (27) | 2.0 [2.0-5.0] | 4.0 [2.0-7.0] | 0.2005 |
| Lethargy (42) | 1.0 [0.0-2.0] | 3.0 [1.0-7.0] | 0.1617 |
| Stereotypy (18) | 0.0 [0.0-1.0] | 2.5 [0.0-5.0] | 0.2023 |
| Inappropriate Speech (12) | 3.0 [1.0-8.0] | 3.0 [2.0-5.0] | 0.9849 |
| Social Avoidance (12) | 0.0 [0.0-6.0] | 4.0 [1.0-7.0] | 0.1919 |
Supplementary Figure S2. Electrode placement and ABCFX domains, including irritability (A), hyperactivity (B), lethargy (C), stereotypy (D), inappropriate speech (E), social withdrawal (F).

## Slide 3
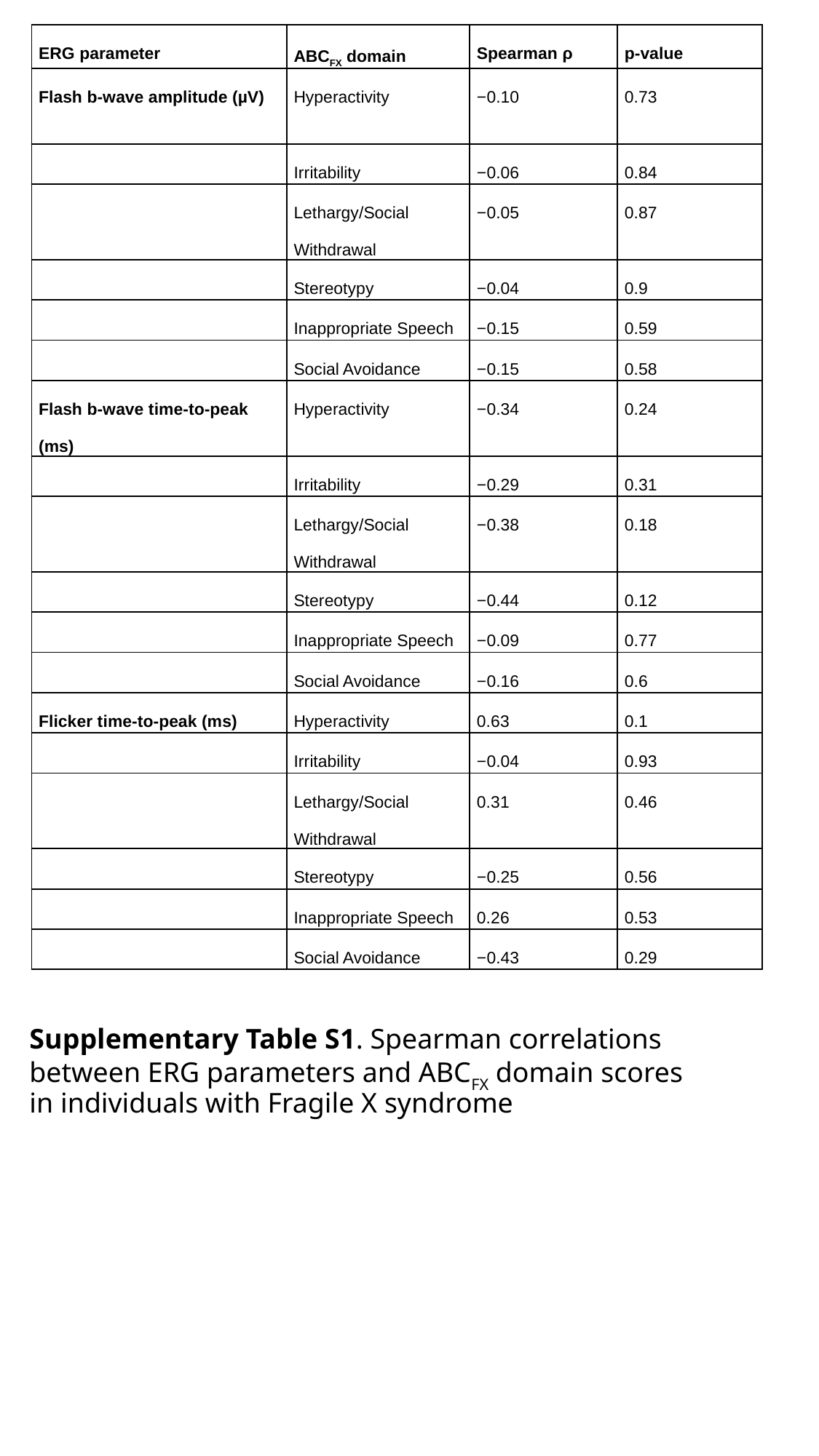

| ERG parameter | ABCFX domain | Spearman ρ | p-value |
| --- | --- | --- | --- |
| Flash b-wave amplitude (µV) | Hyperactivity | −0.10 | 0.73 |
| | Irritability | −0.06 | 0.84 |
| | Lethargy/Social Withdrawal | −0.05 | 0.87 |
| | Stereotypy | −0.04 | 0.9 |
| | Inappropriate Speech | −0.15 | 0.59 |
| | Social Avoidance | −0.15 | 0.58 |
| Flash b-wave time-to-peak (ms) | Hyperactivity | −0.34 | 0.24 |
| | Irritability | −0.29 | 0.31 |
| | Lethargy/Social Withdrawal | −0.38 | 0.18 |
| | Stereotypy | −0.44 | 0.12 |
| | Inappropriate Speech | −0.09 | 0.77 |
| | Social Avoidance | −0.16 | 0.6 |
| Flicker time-to-peak (ms) | Hyperactivity | 0.63 | 0.1 |
| | Irritability | −0.04 | 0.93 |
| | Lethargy/Social Withdrawal | 0.31 | 0.46 |
| | Stereotypy | −0.25 | 0.56 |
| | Inappropriate Speech | 0.26 | 0.53 |
| | Social Avoidance | −0.43 | 0.29 |
Supplementary Table S1. Spearman correlations between ERG parameters and ABCFX domain scores in individuals with Fragile X syndrome

## Slide 4
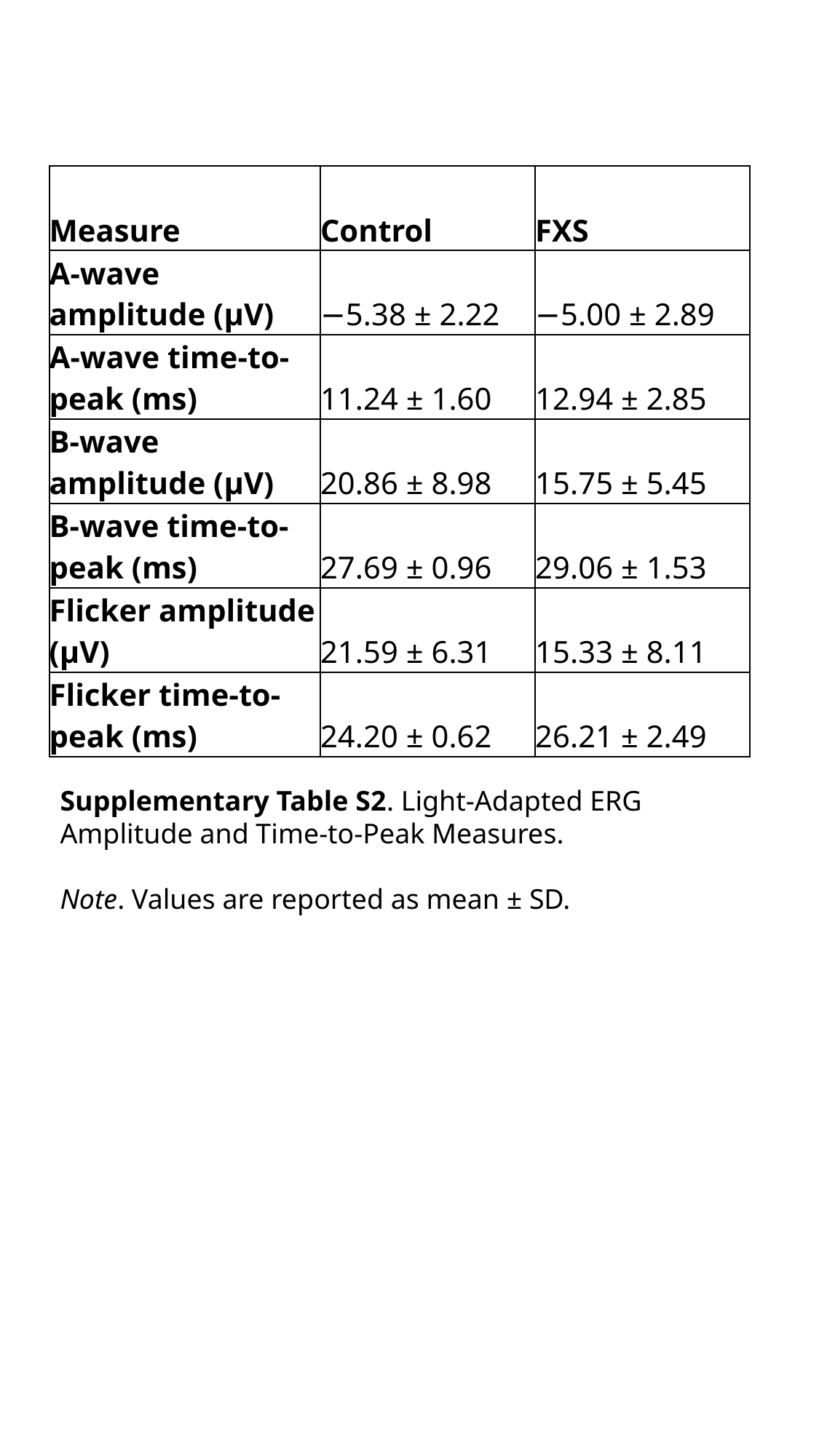

| Measure | Control | FXS |
| --- | --- | --- |
| A-wave amplitude (µV) | −5.38 ± 2.22 | −5.00 ± 2.89 |
| A-wave time-to-peak (ms) | 11.24 ± 1.60 | 12.94 ± 2.85 |
| B-wave amplitude (µV) | 20.86 ± 8.98 | 15.75 ± 5.45 |
| B-wave time-to-peak (ms) | 27.69 ± 0.96 | 29.06 ± 1.53 |
| Flicker amplitude (µV) | 21.59 ± 6.31 | 15.33 ± 8.11 |
| Flicker time-to-peak (ms) | 24.20 ± 0.62 | 26.21 ± 2.49 |
Supplementary Table S2. Light-Adapted ERG Amplitude and Time-to-Peak Measures.
Note. Values are reported as mean ± SD.

## Slide 5
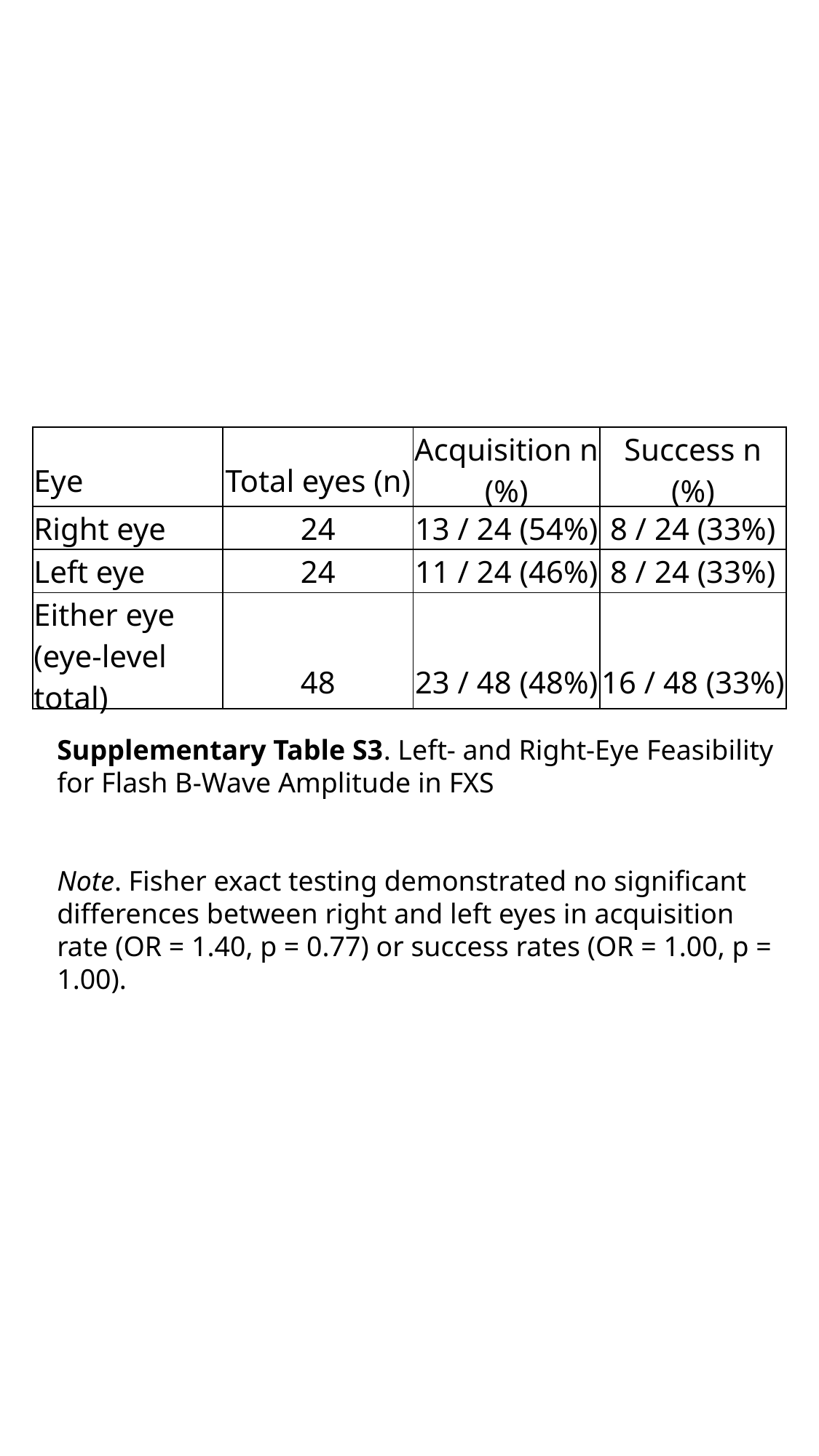

| Eye | Total eyes (n) | Acquisition n (%) | Success n (%) |
| --- | --- | --- | --- |
| Right eye | 24 | 13 / 24 (54%) | 8 / 24 (33%) |
| Left eye | 24 | 11 / 24 (46%) | 8 / 24 (33%) |
| Either eye (eye-level total) | 48 | 23 / 48 (48%) | 16 / 48 (33%) |
Supplementary Table S3. Left- and Right-Eye Feasibility for Flash B-Wave Amplitude in FXS
Note. Fisher exact testing demonstrated no significant differences between right and left eyes in acquisition rate (OR = 1.40, p = 0.77) or success rates (OR = 1.00, p = 1.00).

## Slide 6
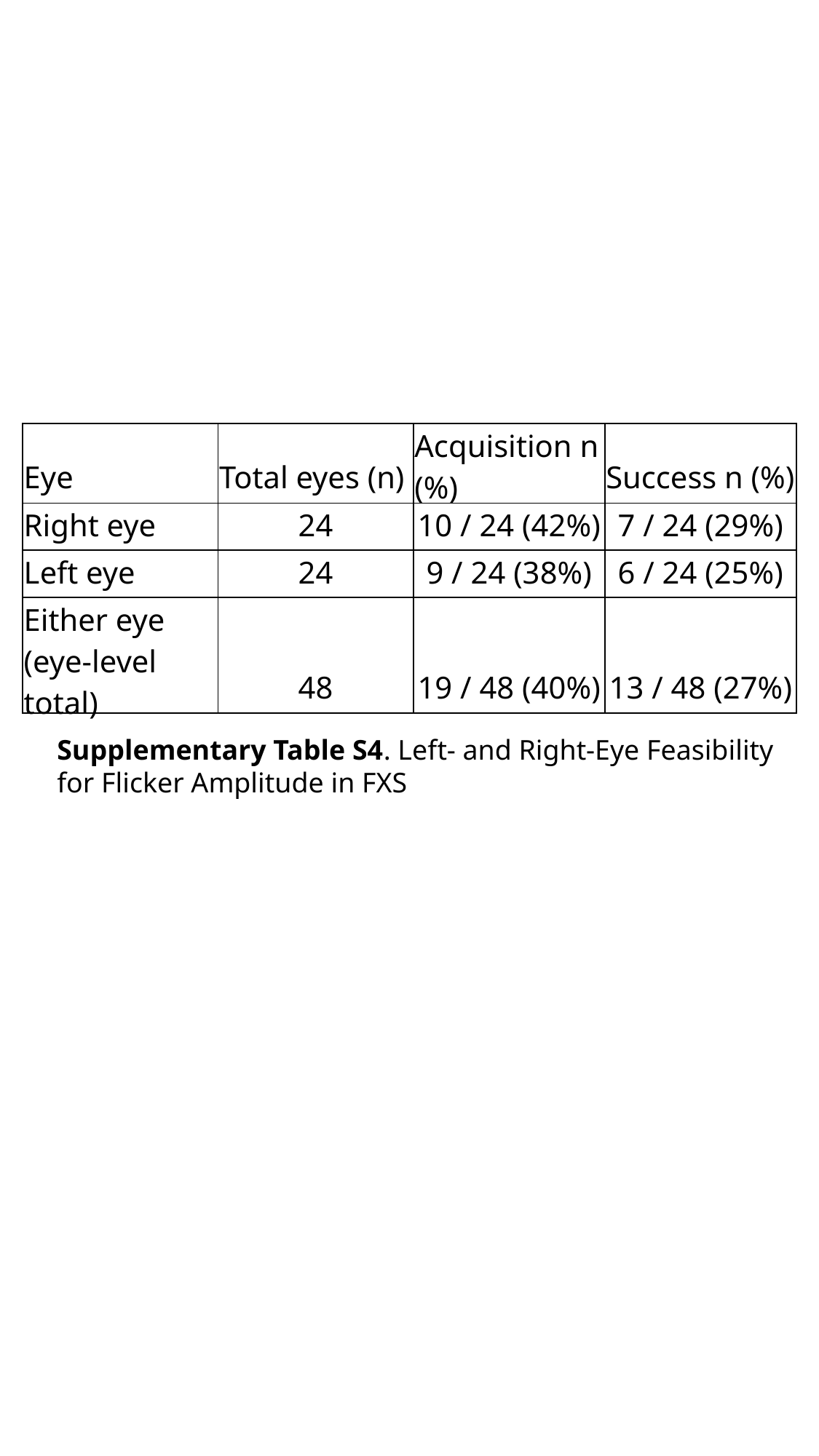

| Eye | Total eyes (n) | Acquisition n (%) | Success n (%) |
| --- | --- | --- | --- |
| Right eye | 24 | 10 / 24 (42%) | 7 / 24 (29%) |
| Left eye | 24 | 9 / 24 (38%) | 6 / 24 (25%) |
| Either eye (eye-level total) | 48 | 19 / 48 (40%) | 13 / 48 (27%) |
Supplementary Table S4. Left- and Right-Eye Feasibility for Flicker Amplitude in FXS
